# From Optoelectronics to Radiation Detection: Light Yield Challenges in Perovskite Scintillators

**DOI:** 10.1021/acsenergylett.5c03188

**Published:** 2025-12-22

**Authors:** Matteo L. Zaffalon, Luca Gironi, Martin Nikl, Sergio Brovelli

**Affiliations:** † Dipartimento di Scienza dei Materiali, 9305 Università degli Studi di Milano−Bicocca, Via R. Cozzi 55, 20125 Milan, Italy; ‡ INFN − Sezione di Milano−Bicocca, 20126 Milan, Italy; § Dipartimento di Fisica, Università degli Studi di Milano−Bicocca, Piazza della Scienza, 20126 Milan, Italy; ∥ Institute of Physics of the Czech Academy of Sciences, Cukrovarnicka 10/112, Prague 16200, Czech Republic

## Abstract

The emergence of nanomaterials, such as lead halide perovskites (LHPs), has catalyzed the development of next-generation scintillators based on thin films and nanocrystals for radiation detection. While these materials offer unique advantages in terms of scalability, emission tunability, and fabrication versatility, accurately quantifying their light yield (*LY*) remains a fundamental yet unresolved challenge. Unlike bulk crystals, thin films suffer from reduced energy deposition, complex optical and luminescence loss mechanisms, and geometry-dependent light extraction—all of which can severely distort *LY* measurements. In this Perspective, we highlight the critical methodological limitations of current *LY* characterization approaches when applied to emerging LHP scintillators. We discuss the theoretical framework underpinning *LY*, the pitfalls of using traditional bulk-based protocols, and the need for precise energy deposition modeling. Finally, we propose practical guidelines for achieving reproducible, accurate *LY* determinations in thin-film or nanocomposite formats, thereby enabling a fair comparison across materials and facilitating the development of high-performance scintillators for emerging radiation detection technologies.

## Graphical abstract

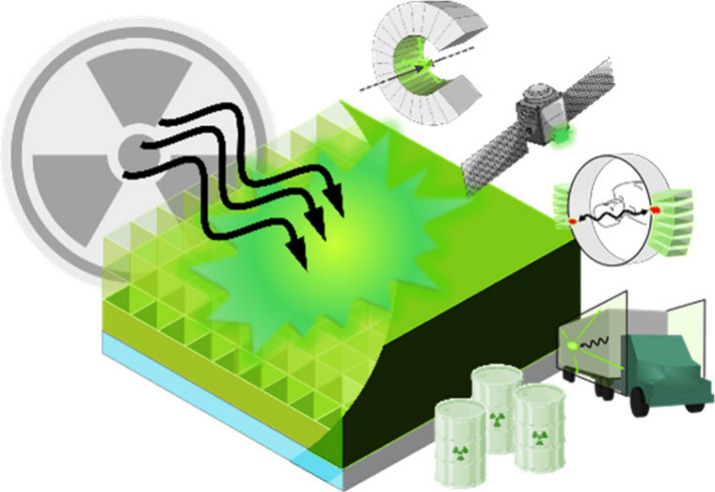

Over the past decades, semiconductor materials produced via wet chemistry and low-temperature deposition have emerged as versatile platforms for scalable, cost-efficient energy technologies.^1^ Their applications span energy generation,^2^ storage,^3^ and low-power optoelectronics, with established roles in photovoltaics,^4^ solid-state lighting^5^ (from displays to high-power LEDs), photocatalytic hydrogen production,^6^ and sensing.^7^

More recently, lead halide perovskites (LHPs) have drawn exceptional attention due to their unique properties, most notably intrinsic defect tolerance,^8^ which suppresses deep trap formation, a longstanding challenge in conventional semiconductors. This enables efficient charge transport in optoelectronic devices and highly efficient excitonic radiative recombination in luminescent applications. Leveraging these properties and extensive insights from nanoscience, LHP-based devices have rapidly progressed toward technological maturity, with promising demonstrations in tandem solar cells,^9^ micro-LEDs for high-definition displays,^10^ low-threshold lasers,^11^ and photodetectors.^12^

In this context, LHPs are emblematic. Progress in *low-energy* applications—such as photovoltaics and photon downshifting in displays—particularly in processing, stabilization, and carrier dynamics control, has laid the foundation for their emerging use in *high-energy* applications, including radiation detection. Traditionally peripheral to the optoelectronics community, this field has now entered the mainstream thanks to LHPs’ high Pb content. While lead is a liability in consumer technologies due to environmental concerns, it is a distinct advantage for ionizing radiation detectors, where efficiency and sensitivity scale with the average atomic number (*Z*) by power laws.^13^ Consequently, LHPs are being actively investigated for large-area X-ray imaging screens,^14^ gamma spectroscopy,^15^ ultrafast time-tagged detection in time-of-flight setups,^16,17^ and, more recently, neutron detection^18^ and space radiation monitoring.^19^ Their favorable elemental composition, combined with unique photophysical properties, has fueled a surge of proof-of-concept demonstrations in both direct (radiation-to-carrier) and indirect (radiation-to-photon via scintillation) detection modes. This is reflected in recent reviews,^20,21^ which testify to the rapid succession of breakthrough performances leveraging surface passivation and controlled deposition strategies.

Thin-film and nanocrystal scintillators demand alternative characterization strategies as conventional light yield methods prove unsuitable.

Yet, the rapid expansion of this field has also exposed a critical gap: the lack of community-wide benchmarks and methodological standards. This is particularly acute in scintillation research, where reliable evaluation of light yield (*LY*)—defined as the number of photons emitted per unit of deposited energy, typically expressed in photons/MeV—is fundamental. Accurate *LY* determination is essential not only for establishing the intrinsic performance of new materials but also for ensuring fair and reproducible comparisons across systems and conditions. Table 1 presents a literature survey of reported *LY* values for various LHP-based materials across different material forms, highlighting the diverse experimental approaches employed.

**Table 1:** Representative *LY* Measurements on CsPbBr_3_-Based Nanoscintillators, Comparing Sample Form, *LY* Measurement Technique, and the Influence of Substrate, Solvent, or Host Matrix^a^

**Feature**	**Composition**	**Sample Form**	**LY (ph MeV** ^**–1**^ **)**	**LY measure Technique**	**Scint. Solvent/Matrix**	**ref.**
**Film**	CsPbBr_3_ NCs	Thin film (20 μm)	21000	Reference scintillator (Ce:LuAG)	N/A	^23^
	CsPbBr_3_/Cs_4_PbBr_6_	Sintered NC powders (0.5 mm thick)	33800	Reference scintillator (LYSO:Ce): sanded crystal 0.6 mm thick	N/A	^24^
	CsPbBr_3_ MH-NCs	Sprayed on paper (20 mg/cm^2^)	30000	Reference scintillator (CsI:Tl)	N	^25^
	CsPbBr_3_ NCs	NCs infiltrated in anodized Al oxide columnar array	11000	Reference scintillator (*LY*SO)	N	^26^
	CsPbBr_3_ NWs	NWs infiltrated in anodized Al oxide columnar array	13200	Reference scintillator (YAG:Ce) + X-ray attenuation correction	N	^27^
	MAPbBr_3_ NCs	Thin film on glass substrate	14600	Reference scintillator (NaI:Tl)	Y	^28^
	CsPbBr_3_ NCs	PVDF substrate + 30 μm NC + Poly styrene coating	16000	Reference scintillator (LuAG:Ce) + X-ray attenuation correction	Y	^29^
	CsPbBr_3_ NCs	Thin film (∼2 μm) on glass substrate	24000	Photoelectric peak excited with ^137^Cs	Y	^30^
	CsPbBr_3_ NCs @ BaF_2_	Wavelength shifter: 8.35 μm film on top of BaF_2_ single crystal	6300	Photoelectric peak excited with ^137^Cs	Y	^31^
**Nanocomposites**	CsPbBr_3_ NCs	PMMA/PLMA nanocomposite (0.8% *w/w*)	4800	Fraction of absorbed X-rays	N	^32^
	CsPbBr_3_ NCs	PMMA nanocomposite (7% *w/w*) 7 mm thick	6000	Reference scintillator (plastic EJ276D) 5 mm thick	N	^33^
	CsPbBr_3_ NCs	Polyacrylate-based composite (200 μm thick 15% *w/w*)	21500	Photoelectric peak excited with ^241^Am	N	^34^
	CsPbBr_3_ NCs	PMMA nanocomposite	15800	Reference scintillator (pressed powders of BGO, CsI:Tl matching shape and volume of the sample)	N	^35^
	ZnS(Ag) decorated with CsPbBr_3_ NCs	Pressed poly ethylene film	40000	Reference scintillator (BGO)	N	^36^
	CsPbBr_3_ NCs + dye	PMMA nanocomposite	9000	Reference scintillator (*LY*SO)	N	^37^
	CsPbBr_3_@Cs_4_PbBr_6_	Film of poly styrene composite ∼20% *w/w*	6000	Reference scintillator (polished CsI:Tl 23.5 × 20 × 0.5 mm)	Y	^38^
	CsPbBr_3_/CsPb_2_Br_5_ NCs	Thin poly styrene composite (40 μm 33% *w/w*)	19200	Reference scintillator (LuAG:Ce)	Y	^39^
	CsPbBr_3_ NCs	PVT nanocomposite (10% *w/w*)	10400	Reference scintillator (plastic EJ276D). RL corrected for self-absorption	Y	^40^
	CsPbBr_3_ NCs in glass-ceramic matrix	Glass matrix	4100	Reference scintillator (BGO)	Y	^41^
	CsPbBr_3_ NWs	SEBS copolymer (∼4% loading) 0.8 mm thick	3800	Reference scintillator (BGO)	Y	^42^
	CsPbBr_3_ NCs/MOF	Poly styrene 25% *w/w* PVK/MOF loading (20 × 20 × 0.2 mm)	24800	Reference Scintillator (CsPbBr_3_ NCs assuming *LY* from other references including ref 38	Y	^43^
	CsPbBr_3_ NCs/MOF	SEBS copolymer, ∼1 mm thick film	∼13500	Reference scintillator (BGO)	Y	^44^
**NC Solutions**	CsPbBr_3_ NCs	Solution—Octane (25 mg/mL)	2300	Reference liquid scintillator (PPO, POPOP in toluene)	N	^45^
	CsPbBr_3_/CsPb_2_Br_5_ NCs	Solution—Water	∼3000	Reference scintillator (PPO in LAB (2 g L^–1^)) + X-ray attenuation correction	N	^46^
	CsPbBr_3_ NCs	NC Solution	24000	Reference scintillator (PEA)_2_PbBr_4_ (*LY* from photopeak measurement)	–	^47^
	CsPbBr_3_ NSs	Solution—Toluene in glass vial	21000	Pulse height spectroscopy with ^137^Cs + Reference scintillator (Ce:LuAG)^a^	Y	^48^
	CsPbBr_3_ NCs + dye	Solution—Toluene (25 mg/mL)	∼16700	Compton edge excited with ^137^Cs + reference liquid scintillator (EJ-301)	Y	^49^
	CsPbBr_3_ NCs + Pyrromethene 580 dye	Solution (10–20% NCs)—trimethylbenzene	∼8500	Compton edge excited with ^137^Cs + reference liquid scintillator (EJ-305)	Y	^50^
**Powders**	CsPbBr_3_ NCs	NC Powders	8500	Reference scintillator (BGO powders)	N/A	^51^
	CsPbBr_3_/Cs_4_PbBr_6_ NCs	NC powders	3600	Reference scintillator (*LY*SO)	N/A	^52^
	CsPbBr_3_/Cs_4_PbBr_6_ NCs	NC powders	64000	Photoelectric peak excited with ^241^Am	N/A	^53^

a We emphasize that the purpose of this table is to highlight the broad spread of LY values reported in the literature, not to validate or endorse any specific measurement or dataset.

b Authors highlight the absence of a clear Compton shoulder, requiring further investigation.

In bulk crystalline scintillators, including single crystal LHPs, *LY* is commonly assessed via pulse height measurements, which involve irradiating crystals with radioisotopes’ gamma-rays and recording the resulting photoelectric peak using coupled photodetectors.^22^ This approach provides estimates of scintillation efficiency and energy resolution, both vital for spectroscopic applications in high-energy and space physics, as well as radiomedical diagnostics. However, the method presumes sufficient radiation stopping power, which bulk crystals naturally provide. Thin-film or nanocrystalline scintillators, by contrast, often lack the density or thickness required to generate detectable photoelectric peaks. These systems, increasingly favored for their scalability, ease of integration, and flexibility thus fall outside the scope of traditional *LY* measurement. Compounding this challenge are altered charge transport and recombination dynamics, along with optical losses from scattering, self-absorption, and inefficient light extraction.

Addressing these limitations requires a thorough, standardized approach to *LY* quantification. Only then emerging LHP scintillators can be reliably benchmarked against established materials and their true potential accurately assessed. This need for methodological awareness is particularly critical for scientists, such as materials scientists, chemists, and solid-state physicists, who enter the field from low-energy optoelectronics, where standardized metrics and protocols are the norm.^54^ A key distinction between scintillation research and fields such as photovoltaics or light-emitting diodes lies in the complexity of measurement. In photovoltaics and LEDs, device characteristics are relatively uniform, allowing the adoption of widely accepted protocols for evaluating parameters such as power conversion efficiency or the external quantum yield of electroluminescence.^55^ By contrast, scintillator detector performance is inseparably tied to both the physical properties of the material (e.g., size, shape, composition, concentration) and the type and energy of the ionizing radiation under study (photons or charged particles).^56^ Since these factors jointly govern the scintillation mechanism and ultimately the *LY*, no single universal experimental protocol can adequately capture the diversity of LHP-based material systems. Instead, accurate *LY* determination requires a careful understanding of the underlying issues and the implementation of tailored experimental strategies that capture the true characteristics of the material under examination.

This perspective highlights the key challenges of *LY* measurement in emerging thin-film, nanocrystal and nanocomposite LHP scintillators and proposes practical guidelines to advance methodological rigor. We outline best practices, common pitfalls, and experimental strategies to enable high-precision, reproducible *LY* measurements and accelerate the development of next-generation scintillator technologies.

Light yield is commonly defined as the number of scintillation photons emitted per unit of deposited energy (conventionally normalized to 1 MeV) under excitation by high-energy photons or particles.^57^ In analogy, photoluminescence quantum yield (*η*_*PL*_) expresses the probability that one photon is emitted for every photon that is absorbed and is typically regarded as an intrinsic material constant. Determining *η*_*PL*_ therefore commonly requires an accurate measurement of optical absorbance (i.e., the number of absorbed photons) and of the resulting photonic output. *LY* is conceptually similar, one must know how much energy *E*_*d*_ is actually deposited and how many photons are emitted, but the practical path diverges sharply. For bulk scintillators, the stopping power is high enough to produce an adequate number of photoelectric events. Hence, both the number of emitted scintillating photons and E_d_ can be inferred from the analysis of the photopeak using pulse-height spectroscopy. Scintillator manufacturers routinely rely on this approach, so that a single *LY* value can be reported without any need to specify sample size or surface finish. This is typical for bulk crystals with negligible self-absorption losses, where light guiding and light outcoupling to the detector are handled by back-diffusing wrappings and index-matching layers, respectively.

Accurate light yield assessment of thin-film nanocrystal scintillators requires tailored experiments addressing material and radiation dependencies.

The absolute measurement of *LY* is typically performed in a gated mode, where only scintillation photons arriving within a defined time window (the so-called shaping time) are counted (thus considering only the “fast” component of the scintillation light) although it can also be assessed in a gateless configuration, such as under steady-state X-ray excitation, where the entire scintillation emission is captured regardless of its temporal profile.^58^ Subsequently, the number of measured photoelectrons in photomultipliers (*N*), or electron–hole pairs in photodiodes, produced by a scintillation light pulse is then corrected for the quantum efficiency of the detection line to estimate the number of emitted photons.^59^ The value of *N* is hence linked to other material and device efficiencies through the expression *N ∝ E*_*D*_ *× η*_*OUT*_ *× η*_*PD*_ *× η*_*SCINT*_, which respectively account for light-outcoupling (*η*_*OUT*_), photodetector (*η*_*PD*_) and scintillation (*η*_*SCINT*_) quantum yields. In turn, *η*_*SCINT*_ depends on the number of electron–hole pairs generated following ionization, energy transport inside the scintillator and *η*_*PL*_ under ionizing conditions. The *LY* can then be expressed as *η*_*OUT*_ *× η*_*SCINT*_. The ideal *LY* of a material can be derived from a simple theoretical model that assumes complete energy deposition and 100% scintillation efficiency: *LY*_*max*_ = *E*_r_/(β × *E*_*g*_) where *E*_r_ is the energy of the incident radiation, *E*_*g*_ is the bandgap of the material, and β is a material-dependent proportionality factor typically in the range 1–3.^60^ For instance, in the case of the perovskite material CsPbBr_3_, with a bandgap *E*_g_ ≈ 2.4 eV and assuming β = 2.5 according to the literature, the theoretical maximum *LY* per 1 MeV absorbed energy can be calculated as *LY*_max_ = 10^6^/(2.5 × 2.4) = 1.7 × 10^5^ photons/MeV. This estimate assumes that the material absorbs the entire energy of the incident radiation and that 100% of that energy is converted into emitted light.

However, the reality is far more complicated. Thin films or low-density nanocomposites (e.g., LHP loading <10% *w/w* in millimeter thick composites) typically absorb only a small fraction of the incident high-energy radiation, as illustrated in Figure 1, and the observable photon output depends not only on the intrinsic conversion efficiency but also on the fraction of energy halted in the sample and on how efficiently the generated photons are transported to the sensor. Hence, what is directly measured is an extensive light output that varies with stopping power, conversion efficiency, and both internal transport and extraction losses. The *LY* itself remains an intensive quantity, but its determination necessarily involves normalizing this extensive signal by the deposited energy and the effective light-collection efficiency, both of which are often poorly constrained when stopping power is low and outcoupling is not fully characterized. As a result, the actual *LY* of thin-film or nanocomposite materials often falls well below its theoretical limit and the challenge in accurately determining its value lies largely in the fact that the energy deposited in the material is often incomplete due to the low stopping power (see Figure 1b). The deposited energy must be carefully accounted for to ensure that accurate *LY* values are obtained.^21,64^ The standard method for measuring *LY* in bulk scintillators, where energy deposition is largely complete, is not directly applicable to thin or low-density scintillators without modifications and adjustments to the experimental protocol. Similarly, relative *LY* measurements performed by comparing the emission intensity to bulk reference crystals is challenging due to the inherent necessity to account for the true stopping power of the sample as well as to optical/geometrical considerations outlined below.

**Figure 1:**
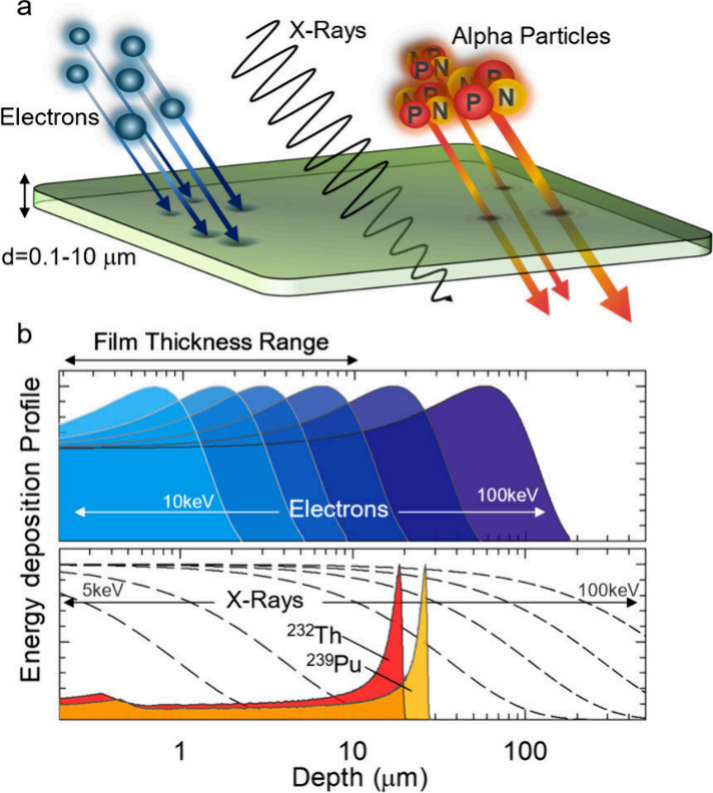
**Energy deposition profiles of α-particles, electrons, and X-rays in a thin film. a)** Schematic illustration of the interaction between high-energy particles or X-rays and a thin scintillating film. Alpha particles emitted from nuclear decay processes typically possess kinetic energies in the 4–10 MeV range. As they slow down within the medium, their energy loss rate increases, culminating in a sharp energy deposition maximum known as the Bragg peak. This peak usually occurs beyond the typical thickness (0.1–10 μm) of thin films. Electrons emitted by electron guns, by contrast, exhibit broader energy deposition profiles but still feature a pronounced maximum. Their kinetic energy can be easily tuned in the keV range, allowing for full energy deposition within the thin film thickness. X-rays, on the other hand, primarily interact via the photoelectric effect and display a long-range exponential attenuation profile. As a result, an X-ray flux tends to deposit only a fraction of its energy within thin films. **b)** Calculated energy deposition profiles in thin films of CsPbBr_3_ (ρ = 4.57 g/cm^3^) for α-particles,^61^ electrons,^62^ and X-rays^63^ at technologically relevant energies. Electron energy deposition is shown in the top panel at increasing energy (*E* = 10, 15, 20, 30, 50, 100 keV). The bottom panel displays α-particles from ^232^Th (4.0 MeV) and ^239^Pu (5.2 MeV), along with X-rays (dashed lines) at various energies (*E* = 5, 10, 20, 30, 50, 100 keV). The deposition profiles for α-particles and electrons were normalized at their maximum to allow for easier visual comparison of Bragg peak depths across different energy scales. X-ray attenuation was calculated using the photoelectric approximation, normalizing the profiles at the initial incident intensity.

Several factors complicate the measurement of *LY* in thin films or nanocrystal-based composites compared to bulk materials. The most significant of these factors are the incomplete energy deposition, reduced/enhanced light extraction efficiency, and the challenges associated with the geometry of thin-film scintillators. In bulk scintillators, the large size of the material ensures that most, if not all, of the incident energy is absorbed. In contrast, thin films and nanocomposites have a much lower stopping power. For example, X-rays, which are commonly used in radiation imaging experiments,^26^ have a penetration depth up to millimeters, whereas the thickness of thin films typically ranges from a submicron (tens or hundreds of nanometers) to tens of microns.

The effect becomes more and more relevant with increasing radiation energy, to the point that measuring the photopeak in pulse height measurements with gamma sources is impossible in most thin films or nanocrystal-based scintillators. This mismatch means that a significant fraction of the energy from the incident radiation passes through the sample without being deposited, leading to a reduction in the measured light output and, in many cases indirectly causing potentially large experimental errors when trying to compensate for such incomplete deposition *a posteriori*. When using polychromatic excitation sources, such as the X-ray tubes commonly found in imaging systems and research laboratories, additional energy and thickness dependencies are introduced due to beam hardening through the film volume (Figure 2). Therefore, caution must be exercised when measuring the *LY* of samples of different thicknesses.

Measuring light yield accurately demands excitation ensuring complete absorption or careful energy deposition quantification.

**Figure 2:**
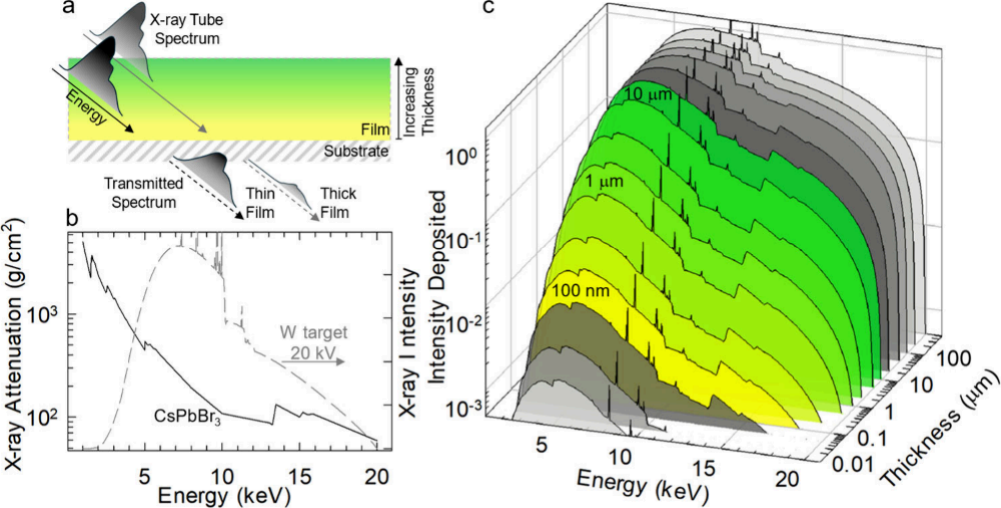
**Attenuation of polychromatic X-rays. a)** Schematic illustration of X-ray attenuation through a thin film and the corresponding hardening of the transmitted spectrum with increasing film thickness. **b)** X-ray attenuation curve^63^ of CsPbBr_3_ as a function of energy (solid black), and a Geant4-simulated X-ray spectrum (dashed gray) from an X-ray tube commonly found in research laboratories, equipped with a tungsten target and operated at 20 kV. **c)** Deposited X-ray intensity calculated after the data shown in (b) for a CsPbBr_3_ film of increasing thickness. The colored region corresponds to typical film thicknesses within the 0.1–10 μm range.

To accurately measure the *LY*, it is thus essential to either use excitation sources that ensure full absorption or to carefully measure or model the fraction of energy deposited in the material.^65^ Simulations, such as Monte Carlo models using Geant4 or other simulation platforms for radiation–matter interaction, can help to predict the energy deposition profile for various radiation types and film geometries, providing critical information for adjusting experimental protocols. Geant4 provides a robust framework for simulating the interaction of ionizing radiation with matter by tracking individual particles through detailed, user-defined geometries. It employs a stepwise Monte Carlo method, where each particle’s trajectory is propagated in discrete steps, allowing for the modeling of energy loss, scattering, and secondary particle production with high spatial and temporal resolution. The toolkit includes an extensive suite of physics models covering electromagnetic, hadronic, and optical processes across a wide energy range. Electromagnetic models simulate phenomena such as ionization, bremsstrahlung, Compton scattering, and pair production, while hadronic models handle elastic and inelastic nuclear interactions using theory-driven and data-based approaches. Optical photon processes, including scintillation and Cherenkov radiation, are also supported. This modular and extensible physics architecture enables precise calculations of energy deposition, interaction cross sections, and particle yields in complex experimental setups. Such simulations, however, are typically limited to the deposition process (by ionizing radiation as well as secondary carriers) and do not explicitly model the scintillation itself, instead relying on experimental parameters, such as optical spectra and scattering estimates, to describe the light outcoupling. For this reason, absolute radiation attenuation measurements are very valuable to practically evaluate the stopping power of real samples. Details of the technique are reported in Table 2. However, for thin films these measurements can be complicated because the contribution of the substrate can represent a substantial, if not the major stopping element of a film-based device (consider that common transparent substrates have thicknesses ranging from hundreds of microns to mm).

**Table 2:** Overview of Key Points of Concern When Evaluating the Parameters Determining the *LY* of Thin Film Scintillators and Recommendations to Address Them^a^

Point of Concern	Need	Sources of Potential Error^b^	Recommendations
**Fraction of Energy Deposited (** *E*_*d*_ **)**	Accurate, quantitative estimation or confirmation of total energy deposition.	1. Partial attenuation in thin films. Size of potential error: *100–1000%.*	1. Select radiation sources that maximize full energy deposition (e.g., low energy X-ray or electron beams).
		2. Increased penetration depth with energy introduces energy and thickness dependence when using nonmonochromatic sources (Figure 3).	2. Perform attenuation measurements using monochromatic sources; report error bars (Figure 4).
			3. Use theoretical simulations (e.g., Geant4).
**Light Outcoupling Efficiency (** *η*_*OUT*_ **)**	Accurate quantitative or comparative estimation.	Varies with material, sample size/shape, and experimental geometry or configuration.	1. Measure *η*_*OUT*_ optically on real-size samples (Figure 5).
		Size of potential error: up to over *200%* (see Figure 2).	2. Choose the most reliable reference sample for comparative light yield (*LY*) measurements
			3. Run Monte Carlo ray-tracing simulations to separate and estimate light scattering, geometric, and reabsorption losses.
**Scintillation Efficiency (** *η*_*SCINT*_ **)**	Accurate quantitative or comparative estimation.	1. Challenges in measuring luminescence yield under ionizing excitation.	1. Conduct absolute radioluminescence (RL) measurements using an integrating sphere and monochromatic excitation to account for *η*_*OUT*_ and *E*_*d*_ (Figures 3, 6a).
		2. Difficulty in quantifying electron/hole pairs generated during transport/thermalization.	2. Select a reliable reference material with a similar emission spectrum and document its physical/chemical characteristics (e.g., type, size, spectra).
		3. Scintillation nonproportionality. Size of potential error: *10–30%*	3. Use calibrated light sources to estimate the spectral response of the optical collection chain and account for detection efficiency inhomogeneities across the reference and sample spectral ranges.
			4. Use several standards with known and different nonproportionality course (e.g., YAP:Ce, BGO, LYSO:Ce). Consider nonproportionality in *LY* if the radiation source used for the *LY* measurement differs from the target application.

a Complement time-resolved RL with time-resolved PL (Figure 6b) to gain qualitative insights into RL vs PL mechanisms (e.g., faster scintillation lifetime may suggest additional nonradiative losses). An instructive example of such additional nonradiative losses due to closely spaced excited states in ionization track is CeF_3_ bulk scintillator.^74^ When dealing with films of quantum confined materials (e.g., colloidal nanocrystals or quantum dots) care should be taken to properly assign kinetic effects to radiative (e.g., multiexciton generation, giant oscillator strength)^17,75^ vs nonradiative (e.g., quenching by excess charges) effects.

b Source of error is meant in situations where inappropriate excitation, standard sample or experiment conditions are used.

**Figure 3:**
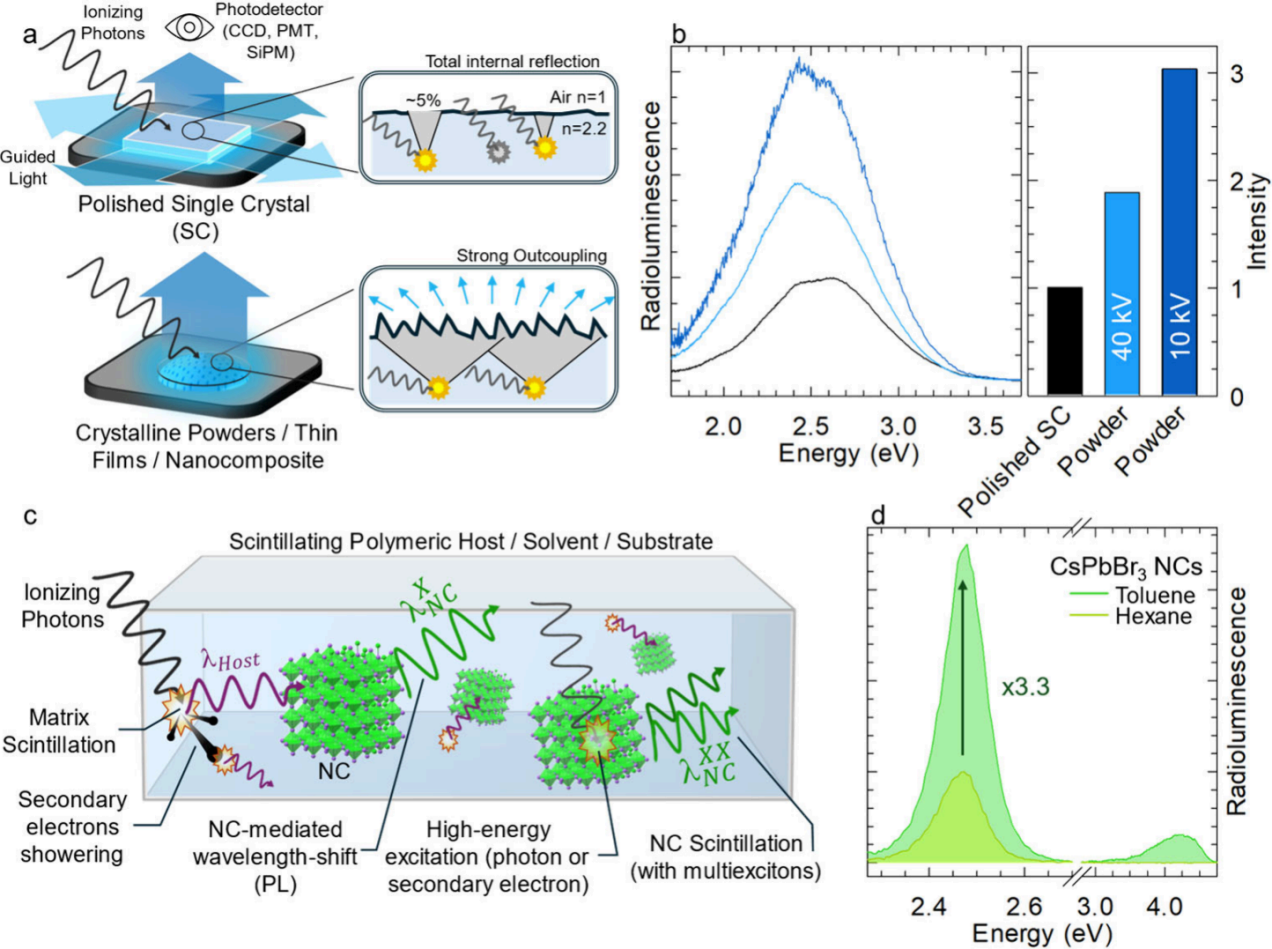
**Light yield (*LY*) determination from radioluminescence (RL) intensity. a)** Schematic illustration of RL measurements performed on a polished single crystal (SC, top) and on a thin film (or, alternatively, crystalline powders or plastic nanocomposites) featuring a rough, as-prepared surface (bottom). A common scenario involves samples thick enough to support geometrical light-guiding effects, resulting in poor light outcoupling from the flat top surface. This is due to total internal reflection, which traps light within the high-refractive-index (*n*) scintillator (e.g., BGO or CsPbBr_3_, *n* ≈ 2.2) in the presence of an abrupt change in refractive index at the surface (without using index matching optical greases); the corresponding light escape cone (gray shaded area) encompasses only ∼5% of the full solid angle. In contrast, surface roughness and volume scattering disrupt waveguiding and internal reflection, enabling more efficient and spatially uniform light extraction. **b)** Representative RL spectra comparing a polished BGO single crystal (black) and its corresponding powders under continuous bremsstrahlung X-ray excitation at 10 kV and 40 kV at the X-ray tube (dark and light blue, respectively). Right panel: extracted RL intensities, normalized to the single crystal response for each excitation energy, reveal up to a 3-fold enhancement in light output from the powder sample. If not properly accounted for, this surface and scattering-induced increase in outcoupling efficiency can lead to overestimations when comparing the relative *LY* of thin films to reference single crystal scintillators. Decreasing voltage at the X-ray tube decreases correspondingly the energy of generated X-rays which are consequently absorbed in thinner sample layer making the luminescence outcoupling more efficient in powder sample while it is rather independent in the single crystal polished plate. **c)** Schematic of scintillation mechanisms in hybrid matrices or solutions containing NCs. When exposed to ionizing radiation, the host matrix (e.g., aromatic polymers/solvents such as PS, PVT, PTP and benzene derivatives, or silicates) produces scintillation light (λ_Host_) typically in the UV spectral region. This host emission can serve as an internal optical pump, exciting the NCs and thereby enhancing the total light yield of the sample. In parallel, NCs may also be excited directly by high-energy photons or by secondary electronic showers generated in the matrix triggering their characteristic multiexciton recombination. **d)** Normalized radioluminescence spectra of CsPbBr_3_ nanocrystal (NC) colloidal solutions prepared at identical NC concentration, dispersed in a nonscintillating solvent (hexane) and a scintillating solvent (toluene). The RL intensity in toluene is ∼3.3 times higher than in hexane, attributable to absorption of toluene’s intrinsic UV scintillation (centered at ∼4.2 eV) and subsequent re-emission by the NCs.

**Figure 4:**
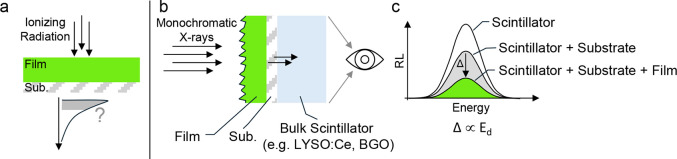
**Method for energy deposition estimation.** **a**) Thin films generally absorb only a small portion of the incident energy, with strong dependence on both thickness and radiation type. **b**) The figure illustrates a straightforward method to estimate the fraction of ionizing energy deposited in a thin film by monitoring the scintillation intensity of a bulk scintillator placed downstream, along the trajectory of the incident radiation. To ensure accurate quantification and avoid artifacts from nonlinear scintillator response or beam hardening, the measurement should be performed using a monochromatic radiation source, such as synchrotron light or emissions from a radionuclide. The bulk scintillator must be sufficiently thick to fully absorb the transmitted radiation and be insensitive to the RL from the film or substrate. When the bulk scintillator is irradiated directly, without any intervening material, it exhibits its maximum RL intensity (**c**). This signal decreases when a bare substrate is placed in front of it (gray curve), further dropping when the substrate is coated with the thin film under investigation (green curve). The difference in RL intensity (Δ) between these two latter configurations is proportional to the energy absorbed by the active film. Note that for polychromatic excitation beams the bulk scintillator might be excited by lower energies when the substrate and substrate + film are inserted, which may introduce nonproportionality effects. As discussed below, this contribution is one to 2 orders of magnitude lower and can be neglected at this level of assessment.

**Figure 5:**
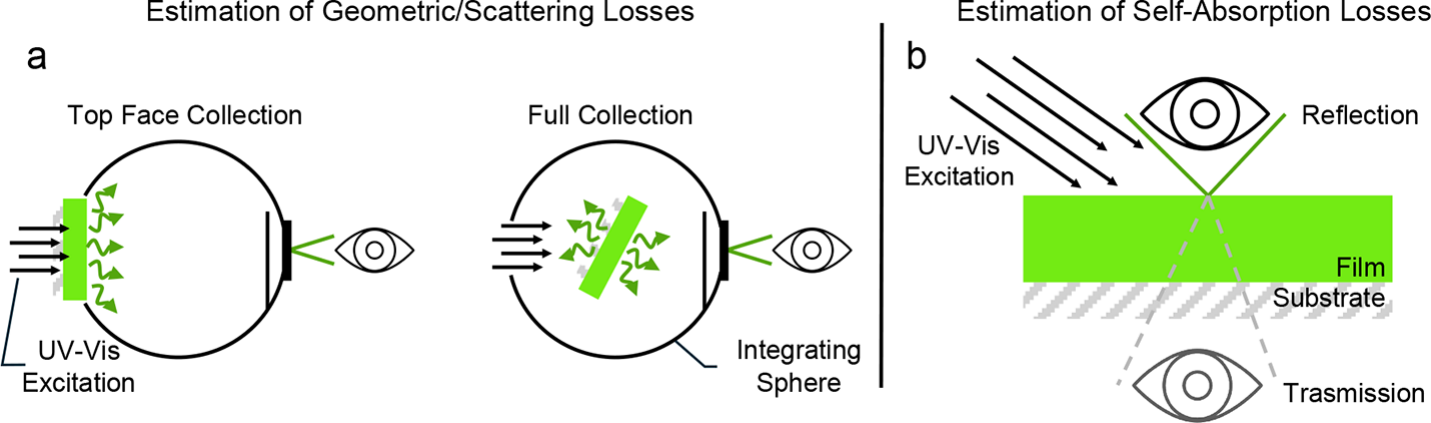
**Light outcoupling and self-absorption effects in thin film. a**) PL measurements using an integrating sphere can be employed to evaluate the impact of surface scattering and light guiding losses in thin films. Comparing the PL intensity collected from the top surface of the film (by facing the film toward the sphere entrance) with the intensity collected from the entire film, including the edges and the back (by placing the film inside the sphere), provides an estimate of light losses and helps to select the most reliable reference sample for comparative *LY*. **b**) Self-absorption losses are critical for efficient light extraction in general, but especially in radiation detection, where the scintillator is designed to interact with radiation throughout its entire volume. This lengthens the light travel inside the scintillator, compared to an equivalent PL measurement where excitation occurs closer to the surfaces. Information useful for guiding the development of new material designs and film processing can be obtained by comparing the luminescence spectra of reflection and transmission configurations, as shown in the diagram. Further information about light guiding inside the film and the substrate can be obtained with the same experimental configuration but collecting the light emitted from the sample sides (i.e., placing the sample with the side edge facing the entrance of the integrating sphere and exciting from the top).

**Figure 6:**
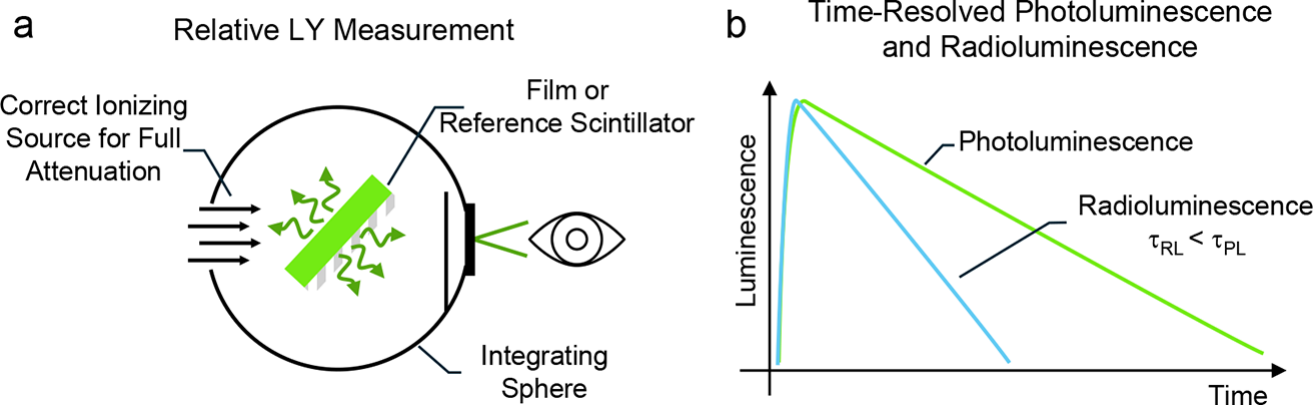
**Scintillation efficiency and complementary insights from time-resolved analysis. a**) The combined use of a suitable radiation source – chosen to be fully attenuated within the thin film – and an integrating sphere for complete RL collection mitigates the impact of differences in outcoupling efficiency (η_OUT_) and effective deposited energy (E_d_) between the thin film and a reference scintillator. **b**) A representative scenario in which RL dynamics appear faster than the corresponding PL, due to the activation of additional loss channels specific to the scintillation cascade. In films of semiconductor nanocrystals, the onset of multiexcitonic processes may be inferred from the emergence of multiexponential decay dynamics (not shown), reflecting distinct excitonic populations.

Another major challenge in thin-film scintillators is accounting for light-extraction efficiency. As noted above, the outcoupling efficiency (*η*_*OUT*_) directly determines the number of photons that escape the material and thus represents a critical parameter for optimizing scintillator performance, for instance, through photonic design strategies such as surface patterning or coupling to optical architectures that enhance and reorient scintillation light. For accurate *LY* measurements, however, differences in *η*_*OUT*_ between reference and test samples pose a serious complication. In bulk scintillators, especially when index matching layers (e.g., oils, greases) are used to couple the crystal to the detector, emitted light can escape comparatively easily. By contrast, thin films often suffer from strong internal light trapping, analogous to planar thin-film light sources when refractive-index mismatch cannot be mitigated by matching media. Because the refractive index of the scintillator is typically much higher than that of the surrounding medium (e.g., air or glass), photons are prone to total internal reflection and tend to propagate laterally toward the film edges, with poor coupling into the photodetector (Figure 3a, b).

At the same time, the surface morphology (i.e., roughness) or the presence of defects/aggregates or different crystalline phases in the bulk act as scattering imperfections that enhance light escape beyond the ideal “cone” factor from highly polished crystals. These effects vary case by case, and influence *LY* measurements performed both with coupled detectors and with remote configurations. Crucially, they are not always evident in conventional measurement protocols yet can introduce significant errors in comparative studies. To address this, methodologies that explicitly account for scattering and optimize light coupling to the photodetector are essential, ensuring reliable *LY* quantification in thin-film scintillators.

Nonradiative recombination is another significant loss mechanism that can reduce the observed *LY*. In thin films, surface effects are often more pronounced as the surface-to-volume ratio is much higher than in bulk materials. These defects can act as nonradiative recombination centers, where energy is dissipated as heat rather than emitted as photons. Additionally, in thin-film systems, the guiding of light along the film plane can lead to the reabsorption of emitted photons. Reabsorption is more likely in materials that emit via direct band-to-band or Wannier exciton electronic transitions or in films thick enough to allow for multiple scattering events, as is the case with lead halides layers commonly used in X-ray imaging. It becomes particularly problematic in materials with low *η*_*PL*_, as a substantial fraction of the reabsorbed light is lost to nonradiative processes. While these loss mechanisms do not complicate the measurement of *LY per se*, it is important to note that the values obtained are dependent on the experimental configuration and may not fully represent the intrinsic behavior of the material itself.

Finally, the characterization of the *LY* of colloidal nanocrystals—particularly LHPs—represents a uniquely delicate challenge that demands particular care. Several factors contribute to this complexity: ***i)*** *Dependence on chemical state and aggregation.* The emission properties of LHP nanocrystals, especially in the absence of specific surface functionalization, are highly sensitive to their chemical environment. Internanocrystal aggregation and energy transfer typically reduce the photoluminescence quantum yield, meaning that *LY* measurements on films may not accurately represent the behavior of isolated particles. ***ii)*** *Effects of ionizing radiation.* Under ionizing radiation, each nanocrystal acts simultaneously as a source of photoelectrons and as a scintillating emitter.^66^ Because the efficiency of secondary excitation (via electromagnetic showers) depends strongly on the interparticle distance,^40,67^ scintillation intensities measured from solid nanocrystal films cannot be directly extrapolated to dilute nanocrystal solutions or nanocomposites and vice versa. ***iii*** **)** *Limitations of dilute-solution measurements.* The “intrinsic” scintillation properties of isolated nanocrystals are more faithfully probed in dilute solutions,^68^ where the interparticle distance exceeds the electron free path. However, these measurements necessarily capture only the response to the very limited energy deposition that occurs within a single nanocrystal. The resulting *LY* is therefore heavily (and nontrivially) dependent on particle concentration, size (which should always be explicitly reported), radiation energy, and aggregation state, making careful control of nanocrystal dispersion essential. These measurements are valuable for investigating the scintillation mechanism but should not be treated as rigid benchmarks of technological validity. Moreover, it is critical to employ nonscintillating solvents or polymeric matrices (typically nonaromatic, such as hexane, cyclohexane, dichloromethane, octane or poly­(methyl methacrylate), polydimethylsiloxane), as scintillating hosts (e.g., toluene, polystyrene, polyvinyl toluene, silica, polyfluorene) introduce indirect excitation pathways through reabsorption and/or energy transfer, leading to *LY* values that reflect the combined emission of solvent and nanocrystals (Figure 3c, d, Table 1). On top of these nanocrystal-specific issues, considerations related to optical light outcoupling described above—such as refractive-index contrast, scattering, and self-absorption—remain fully applicable and can further influence the measured *LY*.

Characterizing light yield of colloidal nanocrystals is a uniquely delicate, careful challenge.

Accurately determining *LY* in thin-film scintillators requires a systematic approach that accounts for the various aspects described above. The following guidelines outline the key considerations for obtaining reliable and reproducible *LY* measurements in thin films.

## Ensure Complete Absorption of Excitation Energy

To accurately measure the *LY*, it is crucial to ensure that the excitation energy is fully absorbed by the material. This can be achieved by selecting radiation sources that are well-matched to the thin-film geometry or to low density samples like dilute solutions. For example, soft X-rays or low-energy electron beams are often used in thin-film scintillator experiments, as they have shorter penetration depths and can be tailored to match the thickness of the films. If full absorption cannot be achieved with a given excitation source, it is important to model or measure the fraction of the energy deposited in the film. This can be done through detailed Monte Carlo simulations or by using dosimetric techniques to measure the actual energy deposition profile of the chosen excitation source within the thin film (Figure 4). The defined energy profile of the excitation radiation is very important. By accurately accounting for the deposited energy, researchers can avoid overestimating the *LY* and ensure that the measurement reflects the true scintillation efficiency of the material. Nonetheless, in order to avoid the need for *a posteriori* correction to the *LY*, the choice of excitation source is one of the most critical factors influencing the reliability of *LY* measurements. A practical concern in *LY* determination is indeed the penetration depth of excitation radiation, especially for thin films. As mentioned previously, alpha particles with energies in the 4–10 MeV range are unsuitable for thin-film scintillators due to their long penetration depths. For example, alpha particles from ^239^Pu are readily available in laboratory settings but have a penetration depth of ∼10–15 μm which is too much for submicron films. Instead, soft X-ray sources (e.g., plasma-based systems as discussed in ref 69) provide attenuation lengths of a few hundred nanometers, making them better suited for thin-layer excitation. Alternatively, cathodoluminescence measurements using low-voltage electron beams (1–10 keV) are ideal for excitation of thin films. These sources can be easily tuned by setting the acceleration voltage at the electron gun to provide the appropriate energy deposition profile for the film’s thickness, ensuring that the energy is deposited well within the material.

## Match Optical Collection and Experimental Conditions

To ensure meaningful comparisons between different materials, it is essential to match the experimental conditions as closely as possible. This includes using the same photodetectors, collection optics, and electronic readout systems for both the sample and the reference material. Additionally, the sample’s size, active area, and positioning relative to the detector must be carefully controlled. Differences in these factors can lead to significant variations in the measured *LY*, even if the materials themselves are similar. Furthermore, the surface morphology of the sample plays a critical role in light extraction. For example, thin-film samples may exhibit different light extraction efficiencies depending on whether they are smooth or rough. The surface finish of the sample should be standardized, or corrections should be applied based on optical models that account for differences in light extraction and scattering (Figure 5). In practice, comparative *LY* measurements should use a reference scintillator with a traceable, well-established *LY* (ideally from pulse-height spectroscopy) that closely reproduces the analyte’s geometry/active area, emission spectrum as seen by the detection chain, morphology (e.g., bulk crystals, powders, plastics, liquids), and surface finish, ensuring that observed differences are attributable to the material rather than to collection geometry or readout. Concrete examples of reference scintillators include polished single crystals (commercially available in multiple thicknesses, ≥50–100 μm) such as LYSO:Ce, LaBr_3_:Ce, and CeBr_3_ as relatively fast, blue-emitting standards, and GAGG:Ce or BGO as green/blue references. When film-like morphology is required, pressed powder beds of bulk crystals or plastic scintillators cut to controlled thickness can better approximate both the stopping power and the light outcoupling of the sample. Importantly, relative RL intensities should be measured under steady-state conditions—readily achieved with a low-ripple continuous ionizing source, preillumination to steady state, and multisecond integrations (*T*_acq_ ≫ τ_scint_). Under these conditions, differences in decay time do not bias the RL intensity, provided any long-lived afterglow or phosphorescence tails (which can extend to minutes in some tungstates^70^) are negligible relative to the integration window.

## Start with Steady-State Radioluminescence Benchmarks

An effective approach for measuring the *LY* of thin films is to begin with steady state radioluminescence or cathodoluminescence measurements. These techniques provide a fast and informative first assessment of the overall scintillation efficiency, without the timing constraints of traditional scintillation measurements with gated detectors.^58^ Steady-state measurements are also less affected by long-lived afterglow or baseline noise, making them ideal for quickly identifying the upper limit of *LY*. Usage of low-afterglow reference materials, such as BGO, for comparison, can help to minimize the baseline uncertainties and provide a stable reference for assessing the *LY* of thin-film samples. While these measurements are not exhaustive, they provide valuable insight into the material’s performance and can guide further, more detailed experiments which might include scintillation kinetics. In this regard, complementing time-resolved RL measurements with the corresponding PL decays offers valuable insight into the distinct mechanisms underlying radio- and photoluminescence, including the activation of nonradiative loss channels or multiexcitonic regimes triggered during the scintillation cascade, both of which may manifest as faster kinetic components.

## Account for Scintillation Nonproportionality

Scintillation nonproportionality refers to the phenomenon where the light output of a scintillator does not decrease proportionally with the decreasing energy of the excitation photons of the incident radiation. This effect is particularly pronounced at low energies below a few tens of keV for electrons and photons, where the material may exhibit significant downward deviations of *LY* because of interaction of elementary excitations due to increasing energy deposit in a material volume unit.^71^ These processes were modeled for various halide materials.^72^ Thin films, due to their surface effects, are particularly susceptible to nonproportionality requiring extra care especially in relative *LY* measurements using a standard scintillating reference. Studies by Dorenbos and others using synchrotron-based monochromatic X-ray excitation down to few hundreds eV excitation energies^73^ have provided valuable benchmarks for understanding the expected scale of nonproportionality in different materials. These studies should be consulted when interpreting the results of *LY* measurements in thin-film scintillators, as they can offer insights into the specific behavior of the material under different excitation conditions.

In summary, the measurement of *LY* in emerging thin-film scintillators presents significant challenges due to incomplete energy deposition, optical and luminescence losses, and surface effects.^21^ These challenges require adopting a systematic approach to *LY* measurement that considers the unique properties of thin films. By ensuring complete absorption of excitation energy, matching experimental conditions when making relative comparison with standard materials, and carefully considering factors such as nonproportionality and light extraction, researchers can obtain accurate and reliable *LY* measurements that truly reflect the performance of thin-film scintillators. Table 2 highlights potential sources of error and suggests approaches to performing correct experimental assessments of the key parameters determining the *LY*. As radiation detection technologies continue to evolve, the importance of accurate *LY* measurements will only grow. Thin-film scintillators hold great potential for a wide range of applications, but their full potential can only be realized when they are characterized accurately and reliably. By adhering to rigorous methodological standards, researchers can ensure that *LY* values accurately capture the capabilities of these new materials, while rigorous reporting will enable meaningful comparisons across laboratories and applications. At minimum, methodological report should: *i*) specify the excitation source (type, energy/spectrum, dose-rate); *ii*) describe how *E*_*d*_ is obtained and its uncertainty (attenuation measurement and/or Monte Carlo simulation; for polychromatic tubes quantify beam hardening and any nonproportionality); *iii*) report any spectral correction applied to the optical detection apparatus when the reference scintillator is spectrally mismatched to the analyte; *iv*) detail the collection geometry, and; *v*) provide sample and reference descriptors (thickness and active area, substrate stack, surface finish/morphology). Adhering to these standards ensures that reported LY reflects the intrinsic performance of thin-film scintillators rather than artifacts of collection or readout.
